# Matrix-assisted laser desorption/ionisation-time of flight mass spectrometry azole susceptibility assessment in *Candida* and *Aspergillus* species

**DOI:** 10.1590/0074-02760220213

**Published:** 2023-03-13

**Authors:** Ana Luisa Perini Leme Giordano, Lais Pontes, Caio Augusto Gualtieri Beraquet, Luzia Lyra, Angelica Zaninelli Schreiber

**Affiliations:** 1 Universidade Estadual de Campinas, Faculdade de Ciências Médicas, Campinas, SP, Brasil

**Keywords:** antifungal susceptibility testing, azoles, MALDI-TOF MS, composite correlation index, Candida, Aspergillus

## Abstract

**BACKGROUND:**

Matrix-assisted laser desorption/ionisation-time of flight mass spectrometry (MALDI-TOF MS) allows rapid pathogen identification and potentially can be used for antifungal susceptibility testing (AFST).

**OBJECTIVES:**

We evaluated the performance of the MALDI-TOF MS in assessing azole susceptibility, with reduced incubation time, by comparing the results with the reference method Broth Microdilution.

**METHODS:**

Resistant and susceptible strains of *Candida* (n = 15) were evaluated against fluconazole and *Aspergillus* (n = 15) against itraconazole and voriconazole. Strains were exposed to serial dilutions of the antifungals for 15 h. Microorganisms’ protein spectra against all drug concentrations were acquired and used to generate a composite correlation index (CCI) matrix. The comparison of autocorrelations and cross-correlations between spectra facilitated by CCI was used as a similarity parameter between them, enabling the inference of a minimum profile change concentration breakpoint. Results obtained with the different AFST methods were then compared.

**FINDINGS:**

The overall agreement between methods was 91.11%. Full agreement (100%) was reached for *Aspergillus* against voriconazole and *Candida* against fluconazole, and 73.33% of agreement was obtained for *Aspergillus* against itraconazole.

**MAIN CONCLUSIONS:**

This study demonstrates MALDI-TOF MS’ potential as a reliable and faster alternative for AFST. More studies are necessary for method optimisation and standardisation for clinical routine application.

Fungal pathogens can cause a wide range of diseases in humans, from easy-to-treat superficial skin and mucous membrane infections to life-threatening invasive infections.^1^ ^,^ ^2^ Invasive fungal infections (IFI) are associated with high mortality rates and patient survival increases with rapid diagnostic and early initiation of appropriate antifungal therapy.^3^ *Candida* species are the most common pathogens causing IFI, while *Aspergillus* genera appear as the most prevalent mold.^4^ ^,^ ^5^ Azoles drugs [*e. g.*, fluconazole (FLZ), itraconazole (ITC), and voriconazole (VRC)] are recommended for aspergillosis treatment and are widely used to treat candidiasis.^3^

The current use of antifungals raises a concern about the selection of resistant strains and their dissemination potential. *Candida* and *Aspergillus* azole resistance represent an emergent clinical challenge.^6^ Azole drugs target the cytochrome P450 14-alpha-demethylase enzyme, which is required for the conversion of lanosterol into ergosterol and is encoded by the ERG11 gene in yeasts and the Cyp51 gene in filamentous fungi. Point mutations and/or overexpression in the referred genes, and other mechanisms (such as mutations in transcriptional regulators, calcineurin, and others) are associated with azole resistance.^3^ ^,^ ^7^

The Clinical and Laboratory Standards Institute (CLSI) and the European Committee on Antibiotic Susceptibility Testing (EUCAST) indicate broth microdilution (BMD) as the reference method for antifungal susceptibility test (AFST). This method is based on microorganism growth evaluation for determining the minimal inhibitory concentration (MIC), which predicts the therapeutical efficacy of the tested drug.^3^ ^,^ ^8^ The BMD method is generally restricted to reference laboratories since it requires trained professionals for performing both the technique and result interpretation. MIC determination is subjective since it requires visual reading of each drug concentration to compare microorganism growth.^9^ ^,^ ^10^ ^,^ ^11^ Besides, the procedure is laborious and time-consuming, hindering the early diagnosis of IFI.^4^ Given those limitations, there is a need for alternative AFST methods that are equally robust, but faster, easy to perform, and able to give objective result interpretation.^12^ ^,^ ^13^

Matrix-assisted laser desorption ionisation-time of flight mass spectrometry (MALDI-TOF MS) technique is a well-established method for rapid and accurate pathogen identification through its protein profile. Besides microbial speciation, the MALDI-TOF MS system has also been applied for antimicrobial susceptibility assessment.^9^ ^,^ ^12^ The main advantages of the antifungal susceptibility test by MALDI-TOF MS (AFST-MS) are the time-to-result reduction and subjectivity elimination in result interpretation.^4^ ^,^ ^9^ Numerous methods employing MALDI-TOF MS to access antibiotic susceptibility in bacteria are already described,^14^ while two main methods are well described for AFST-MS: based on the composite correlation index (CCI) tool of MALDI Biotyper (Bruker Daltonics) system, and on the semi-quantitative approach MBT-ASTRA, which was primarily developed for bacterial applications.^9^ ^,^ ^14^ The main focus of AFST-MS studies reported thus far relates to yeast susceptibility assessment,^15^ while studies investigating molds are still scarce.^16^ ^,^ ^17^

The CCI tool approach for AFST-MS is based on the drug-induced protein composition change that occurs when microorganisms are exposed to different concentrations of the tested antifungal^4^ and can be characterised by MALDI-TOF MS, enabling the inference of the minimal profile change concentration (MPCC) breakpoint. The MPCC is defined as the lower drug concentration that alters the microorganism protein profile^15^ and was shown to be analogous to MIC in previous studies, providing correct microorganism susceptibility assessment.^15^ ^,^ ^16^ ^,^ ^17^ ^,^ ^18^

This study aims to evaluate the CCI-based MALDI-TOF MS method for the assessment of *Candida* spp. and *Aspergillus* spp. azole susceptibility, with reduced incubation time, by comparing the results with the gold standard method Broth Microdilution.

## MATERIALS AND METHODS

*Ethical considerations* - The present study was approved by the local Ethics Committee (CAAE 02286718.0.0000.5404).

*Isolates* - Thirteen resistant and susceptible *Aspergillus* spp. (8 *A. fumigatus*, 1 *A. flavus*, 1 *A. oryzae*, 1 *A. terreus*, 1 *A. alabamensis* and 1 *A. lentulus*) isolates and thirteen *Candida* spp. (6 *C. albicans*, 3 *C. tropicalis* and 4 *C. parapsilosis* complex), obtained from different clinical specimens, and the reference strains *A. fumigatus* ATCC 204305, *A. flavus* ATCC 204304, *C. albicans* ATCC 90028, and *C. parapsilosis* ATCC 22019, were analysed in this study.

The microorganisms were retrieved from the Laboratory of Fungal Investigation (LIF) strain collection in the State University of Campinas Clinical Hospital, subcultured in Sabouraud Dextrose Agar and incubated at 35**º**C until growth was sufficient.

*Aspergillus* species were confirmed by comparative DNA analyses of betatubulin (β-tubulin 2A/-B) sequences, and the calmodulin genes for the *Aspergillus* section *Flavi*,^19^ ^,^ ^20^ *Candida* speciation was confirmed by MALDI-TOF MS, using the Biotyper 3.1 software.

*Broth microdilution* - MIC was determined according to CLSI M38-A2 guidelines for *Aspergillus* ^21^ and M27-A3 guidelines for *Candida* ^22^ isolates using prepared plates (Eiken Chemical Co., Tokyo, Japan). *Aspergillus* spp. was evaluated against ITC and VRC and *Candida* spp. against FLZ.

After fungal growth in Sabouraud Dextrose Agar, the inoculum was prepared in 5 mL of 0.85% saline solution. For *Candida* strains, the inoculum density was adjusted according to McFarland’s 0.5 scale using a spectrophotometer at 530 nm. For *Aspergillus*, the inoculum was obtained to a final concentration of 0.5 to 2.5x10^4^ CFU/mL by counting the conidia in the central reticulum of a Neubauer chamber. The serial dilutions of the antifungals are adsorbed at the bottom of the plate wells. FLZ were evaluated in the concentration range of 0.12-64 µg/mL and ITC and VRC in the concentration range of 0.015-8 µg/m.

For the tests, 100 µL of RPMI culture medium containing the inoculum at a final concentration of 0.5-2.5 x 10^3^ colony forming units per mL for *Candida* and 0.5-2.5 x 10^4^ CFU/mL were added for *Aspergillus*, including the positive control column. In the negative control column, 100 µL of RPMI was added. The plates were incubated at a temperature of 35**º**C, and the MIC reading was performed after 24 and 48 h of incubation for *Candida* and *Aspergillus*, respectively.

*AFST-MS* - AFST-MS was conducted as described by De Carolis et al. with modifications.^17^ The inocula were adjusted to 107 CFU/mL and cultivated in Mueller Hinton Broth containing serial dilutions of antifungal drugs (0.12-64 µg/mL for FLZ; 0.015-8 µg/mL for ITC and VRC) and a null-drug control, in a final volume of 300 µL. The same antifungal concentrations used in BMD were applied to properly compare the different AFST methods. After incubation of the yeasts and moulds for 15 h at 35ºC with constant rotation, each suspension was centrifuged at maximum speed for 2 min, followed by two washes with 1 mL of deionised water and one with 1 mL of 75% ethanol. For protein extraction, the cell pellet was dried at room temperature, suspended in 70% formic acid (20 to 100 µL) and the same amount of acetonitrile (20 to 100 µL), and centrifuged again. One µL of the supernatant was spotted on the MALDI plate and dried at room temperature. Finally, was added one µL of matrix solution, composed of α-cyano-4-hydroxycinnamic acid in 50% acetonitrile-2.5% trifluoroacetic acid, and incubated at room temperature until complete drying.

Protein spectra were acquired with Microflex LT^®^ (Bruker Daltonics, Germany/USA) equipment, and visualised by FlexControl™ software (Bruker Daltonics, Bremen, Germany).

*Data analysis* - The protein spectra of isolates exposed to 10 different drug concentrations and a null-drug control was acquired and imported to Biotyper™ 3.0 software (Bruker Daltonics*,* Bremen, Germany). It was used the CCI statistical tool, as described by De Carolis et al.,^17^ which analyses variations between acquired protein spectra from different drug concentrations. The level of the correlation between spectra and autocorrelations was numerically determined. CCI values close to 1 indicate high similarity and CCI close to 0 indicate low similarity. To facilitate the visualisation of all results, they were automatically translated to a heat map, where closely related spectra are marked in warm colours (dark red to yellow) and a low correlation between spectra is marked in cold colours (dark blue to light green). This allows the visualisation of where there is a protein profile alteration - the MPCC - by observing the similarity degree of protein spectra acquired from the different drug dilutions between each other and between the two extreme conditions (null-drug and maximum drug concentration).

## RESULTS

The reference method indicates that eight (53.33%) molds were susceptible to ITC, four (26.66%) resistants, and three (20.00%) categorised as *Insufficient Evidence* (IE), which means that there is not enough evidence to indicate that the organism or group can be categorised as susceptible or resistant for therapy against the tested antifungal.^23^ Five (33.33%) molds were susceptible to VRC, two (13.33%) resistants, six (40.00%) categorised as IE and two (13.33%) belonging to the *Area of Technical Uncertainty* - interpretation to MICs that cannot be categorised without additional information indicating to the laboratory that a decision referring the treatment conduction has to be made.^23^ Thirteen (86.66%) yeasts were susceptible to FLZ and two (13.33%) were resistant. Susceptibility breakpoints were established following the CLSI M27-A3 guidelines for *Candida* spp. and the EUCAST 10.0 (2020) document^23^ for *Aspergillus* spp., as the breakpoint values for these molds are not established in the CLSI M38-A2 document.^21^

AFST results obtained by BMD and AFST-MS are exposed in Table. Results were considered concordant according to Espinel-Ingoff et al., which state that the cut-off of ± two dilutions is the maximum discrepancy accepted for the definition of the agreement between two different AFST methods.^24^ The overall agreement between methods was 91.11%, for both *Aspergillus* and *Candida* isolates after 15 h of incubation. When results are analysed based on the tested antifungals, 100% of agreement was shown for *Aspergillus* against VRC and *Candida* spp. against FLZ; and a 73.33% agreement rate was reached for *Aspergillus* sp. against ITC. Non-concordant results are marked in bold and only occurred in *Aspergillus fumigatus* ITC resistant strains, for which all the presented MIC was > 8 μg/mL. Nevertheless, even for those cases, the breakpoint presented by AFST-MS detected the reduced susceptibility of those strains.

**TABLE:** Comparison between antifungal susceptibility testing (AFST) by Broth Microdilution and matrix-assisted laser desorption/ionisation-time of flight mass spectrometry (MALDI-TOF MS) methods

nº LIF	Identification	Drug tested	Broth microdilution		MALDI-TOF MS
			MIC (µg/mL)	Susceptibility	MPCC (µg/mL)
2597	*A. fumigatus*	itraconazole	1	S^*a*^	0.5
		voriconazole	1	S	0.25
2598	*A. fumigatus*	itraconazole	0.5	S	0.5
		voriconazole	1	S	0.25
2602	*A. fumigatus*	itraconazole	1	S	0.25
		voriconazole	1	S	1
2664	*A. oryzae*	itraconazole	0.5	IE^*b*^	2
		voriconazole	1	IE	1
2596	*A. flavus*	itraconazole	0.5	S	0.5
		voriconazole	1	IE	1
2486	*A. terreus*	itraconazole	0.25	S	0.12
		voriconazole	1	IE	0.25
2513	*A. alabamensis*	itraconazole	0.5	IE	0.25
		voriconazole	1	IE	0.5
2546	*A. fumigatus*	itraconazole	0.5	S	2
		voriconazole	1	S	0.25
2354	*A. lentulus*	itraconazole	0.5	IE	2
		voriconazole	8	IE	4
2328	*A. fumigatus*	itraconazole	> 8	R^*c*^	4
		voriconazole	8	R	2
2552 4.9	*A. fumigatus*	itraconazole	> 8	R	2
		voriconazole	2	ATU^*d*^	2
2444.6	*A. fumigatus*	itraconazole	> 8	R	2
		voriconazole	2	ATU	2
263-e	*A. fumigatus*	itraconazole	> 8	R	4
		voriconazole	4	R	2
15355	*C. albicans*	fluconazole	1	S	0.5
12560	*C. albicans*	fluconazole	8	R	8
14447	*C. parapsilosis* complex	fluconazole	0.5	S	0.5
14451	*C. parapsilosis* complex	fluconazole	0.5	S	0.25
14529	*C. tropicalis*	fluconazole	0.25	S	0.25
14846	*C. tropicalis*	fluconazole	0.5	S	1
14853	*C. parapsilosis* complex	fluconazole	0.5	S	1
15134	*C. parapsilosis* complex	fluconazole	0.5	S	2
15188	*C. albicans*	fluconazole	1	S	0.5
15234	*C. albicans*	fluconazole	0.5	S	0.5
15272	*C. albicans*	fluconazole	1	S	1
15292	*C. tropicalis*	fluconazole	0.5	S	1
E-10	*C. albicans*	fluconazole	64	R	64
*A. flavus* ATCC 201304		itraconazole	0.5	S	0.5
		voriconazole	1	IE	1
*A. fumigatus* ATCC 204305		itraconazole	0.5	S	1
		voriconazole	0.5	S	1
*C. albicans* ATCC 90028		fluconazole	0.5	S	0.5
*C. parapsilosis* ATCC 22019		fluconazole	2	S	2

LIF: Laboratory of Fungal Investigation; MIC: minimal inhibitory concentration; MPCC: minimal profile change concentration; *a*: susceptible; *b*: insufficient evidence that the organism or group is a good target for therapy with the agent; *c*: resistant; *d*: area of technical uncertainty.

By analysing the resistant strains individually, the *A. fumigatus* isolates LIF 2552-4.9 (MN684334) (ITC MIC: > 8 μg/mL, VRC MIC: 2 μg/mL) and LIF 2444.6 (MN684333) (ITC MIC: > 8 μg/mL, VRC MIC: *2* μg/mL*)* carry *CYP*51A TR34/L98H/S297T/F495I mutation, reported by our group in Pontes et al. (2020);^25^ LIF 263-e (ITC MIC: > 8 μg/mL, VRC MIC: 4 μg/mL) is an environmental isolate that has the mutation in the *CYP*51A TR46/F495I gene (data not yet published); LIF 2328 (ITC MIC: > 8 μg/mL, VRC MIC: 8 μg/mL) did not present mutation in *CYP*51A gene, having their resistance mechanism still not elucidated. The isolate LIF 2354 (ITC MIC: 0.5 μg/mL, VRC MIC: 8 μg/mL) refers to an *A. lentulus*, cryptic specie from the *Fumigati* section, considered intrinsically resistant to azole drugs.^26^ The *C. albicans* isolates LIF 12560 (FLZ MIC: 8 μg/mL) present the amino acid substitution E116D, T128K, E266D, and A298V in the ERG11 gene; LIF-E10 (FLZ MIC: 64 μg/mL) present G448V and G464S substitution in ERG11 gene, these strains were reported by our group in Peron et al. (2016).^27^ It is important to note that the reduced susceptibility was detected by MALDI-TOF MS regardless of the resistance mechanism, even in the few cases in which the discrepancy between the methods exceeded two dilutions.

**Fig. 1:**
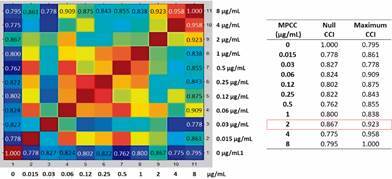
heat map of Laboratory of Fungal Investigation (LIF) 2444.6 (*Aspergillus fumigatus*) against itraconazole (ITC). After 15 h exposure to serial dilutions of ITC, protein spectra were acquired and used to generate the composite correlation index (CCI) matrix. Similar spectra are marked in warm colours and non-related spectra in cold colours. The same ITC concentrations are gathered on the x and y-axis. Spectras above the minimal profile change concentration (MPCC) show similarity with the maximum drug treatment. MPCC = 2 µg/mL. Source: Biotyper™ 3.0 (Bruker Daltonics, Bremen, Germany).

**Fig. 2:**
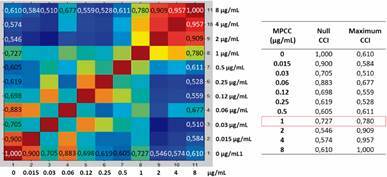
heat map of Laboratory of Fungal Investigation (LIF) 2602 (*Aspergillus fumigatus*) against voriconazole (VRC). After 15 h exposure to serial dilutions of VRC, protein spectra were acquired and used to generate the composite correlation index (CCI) matrix. Similar spectra are marked in warm colours and non-related spectra in cold colours. The same VRC concentrations are gathered on the x and y-axis. Spectras above the minimal profile change concentration (MPCC) show similarity with the maximum drug treatment. MPCC = 1 µg/mL. Source: Biotyper™ 3.0 (Bruker Daltonics, Bremen, Germany).

**Fig. 3:**
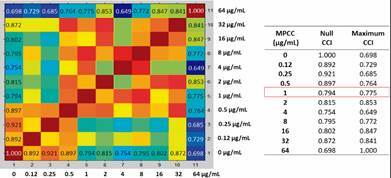
heat map of Laboratory of Fungal Investigation (LIF) 15292 (*Candida tropicalis*) against fluconazole (FLZ). After 15 h exposure to serial dilutions of FLZ, protein spectra were acquired and used to generate the composite correlation index (CCI) matrix. Similar spectra are marked in warm colours and non-related spectra in cold colours. The same FLZ concentrations are gathered on the x and y-axis. Spectras below the minimal profile change concentration (MPCC) show similarity with the control treatment (null drug). MPCC = 1 µg/mL. Source: Biotyper™ 3.0 (Bruker Daltonics, Bremen, Germany).

After protein spectra acquisition of the microorganism exposed to all different drug concentrations, the CCI matrix was generated and all CCI numbers were then automatically translated into a heat map. For analysis illustration, here we present the results of the heat map (on the left), the CCI values (on the right) of the two extreme conditions, and the MPCC value of the isolates LIF 2444.6 (*A. fumigatus*) against ITC, LIF 2602 (*A. fumigatus*) against VRC and LIF 15292 (*C. tropicalis*) against FLZ, in Figs 1, 2 and 3, respectively.

## DISCUSSION

Late diagnosis, even for a few hours, raises the morbidity and mortality rates of patients with IFI and can lead to inappropriate antifungal therapy. In this context, MALDI-TOF MS plays an important role in reducing hospitalisation, leading to a better prognosis in intensive care unit patients.^28^ ^,^ ^29^ ^,^ ^30^ Furthermore, poorer outcomes were found in patients infected by resistant strains than in those infected by susceptible strains.^16^ ^,^ ^31^ Resistant fungal pathogens have been reported worldwide, especially azole resistance among *C. albicans* and *A. fumigatus* isolates, which can result in therapeutical failures. Considering the above-mentioned, AFST has become increasingly relevant in clinical practice.^3^ ^,^ ^9^ ^,^ ^16^

Many studies emphasise the importance of determining fungal pathogens’ susceptibility profile, not only for the appropriate antifungal selection but also to monitor the eventual emergence of resistant strains during the treatment.^3^ ^,^ ^32^ ^,^ ^33^ In the case of *Aspergillus* genera, AFST is not commonly performed in clinical routine, which incurs an underestimation of the resistance incidence.^32^ ^,^ ^33^ Besides the reference method BMD, there are other available AFST techniques, already reviewed in Posteraro et al.,^12^ as commercial methods (*e.g.*, manual assays like Sensititre™, YeastOne™, E-test^®^, and automated means as VITEK^®^ 2 system), aside from molecular detection of resistance genes.^15^ ^,^ ^34^ The current methods have long turnaround times, making clinical mycology in need of faster and more objective techniques.^15^ ^,^ ^33^

As a robust analytical technique for microbial protein detection, MALDI-TOF MS revolutionised clinical laboratories’ routines by promoting rapid, accurate, and cost-effective pathogen identification. The possibility of applying this technology for antimicrobial susceptibility assessment has been investigated and was shown to be a promising technique.^12^ ^,^ ^15^ Few studies evaluate MALDI-TOF MS for AFST, especially in analysing molds.^9^ ^,^ ^35^ The present investigation explored MALDI-TOF MS potential in assessing the azole susceptibility of the major yeast and mold genera related to IFI, *Candida* spp., and *Aspergillus* spp., respectively.^4^ ^,^ ^5^

To the best of our knowledge, ten studies have been reported regarding the application of the same approach for AFST-MS as our present work: the CCI-based method.^4^ ^,^ ^10^ ^,^ ^16^ ^-^ ^18^ ^,^ ^36^ ^-^ ^40^ Among them, nine evaluated the method to assess the susceptibility of different *Candida* species against azole and echinocandins,^15^ ^,^ ^40^ and only two tested *Aspergillus* species. De Carolis et al. applied the method to assess the susceptibility of six *A. fumigatus* and four *A. flavus* isolates against caspofungin;^17^ and Gitman et al. assessed VRC susceptibility in seventeen *A. fumigatus*, two *A. ustus*, and one *A. calidoustus* isolate,^16^ both authors reached full agreement with BMD. The present work is the first to use CCI-based AFST-MS to assess ITC antifungal susceptibility, and also the first to include the *Aspergillus* species *A. lentulus*, *A. terreus*, *A. alabamensis*, and *A. oryzae*.

Among the aforementioned reports, authors have described abbreviated versions of the CCI-based approach. Vella, De Carolis, and co-authors shortened the incubation time to 3 h and simplified the ICC matrix with three antifungal concentrations. This method was evaluated with *C. albicans*, *C. glabrata* and *C. auris* strains against caspofungin, anidulafungin, and FLZ. Susceptibility of more than 90% of isolates was correctly assessed, exhibiting partial agreement by evaluating *C. glabrata* against anidulafungin (25-100% of accuracy, depending on strain resistance mechanism).^18^ ^,^ ^38^ ^,^ ^40^ Paul et al. reached 100% of agreement with BMD by evaluating *C. tropicalis* exposed to serial dilutions of FLZ for 4 h.^35^ Roberto et al. evaluated *C. parapsilosis* complex with 3 h exposition to serial dilutions of the three echinocandins reaching 95%-100% of agreement with BMD.^4^ Delavy et al. evaluated *C. albicans* exposed to three different FLZ concentrations, and with or without cyclosporin, for 3 h; reaching general accuracy of 85.71% regardless of the tolerance phenomenon.^39^

Despite the drastic time-to-result reduction described above, the tests have been conducted with *Candida* species, that generally grow faster. For this first evaluation, we opted for 15 h of incubation, mostly due to the *Aspergillus* species inclusion, especially cryptic species that are known to grow slowly, like *A. lentulus*.^41^ We also opted for the extended method, exposing the microorganisms to serial dilutions (10 different antifungal concentrations and a null-drug control), for better comparison with the gold-standard method, allowing agreement determination according to Espinel-Ingroff et al.^24^ Due to the evaluation of strains against three different drugs, with eleven different drug concentrations for each strain, we did not include a larger number of isolates in the present work, which represent a limitation of the study. Furthermore, we did not have a larger number of resistant isolates cases in our clinical laboratory.

Our group have also performed tests using the RPMI-1640 medium to prepare the inocula. The Mueller Hinton liquid media proved to be more efficient for AFST-MS, especially in the case of filamentous fungi. When using the RPMI-1640 medium, the microorganisms tended to attach to the tube wall, hindering the recovery of the biological material along the washes, of times resulting in insufficient peaks to generate protein spectra. For this reason, we carried out the tests using Mueller Hinton broth.

We demonstrate that it was possible to obtain concordance between AFST-MS and BMD method, reaching an overall agreement rate of 91.11%. Full agreement (100%) between methods was observed in the susceptibility determination of *Aspergillus* spp. against VRC and *Candida* spp. against FLZ. However, by analysing *Aspergillus* spp. against ITC, the agreement rate decreased to 73.33%. There were in total four discrepant cases, all of them occurring with *A. fumigatus* isolates tested against ITC, showing a BMD MIC of > 8 μg/mL. It is important to highlight that BMD measures susceptibility by detecting microorganism growth, while AFST-MS measures susceptibility by detecting proteome modification in the presence of the antifungal.^4^ ^,^ ^15^ ITC MPCCs of the discrepant cases variated between 2 and 4 μg/mL, which still indicates the isolates as having reduced susceptibility. Despite the lack of phenotypic change of those isolates (presenting growth in the presence of all different ITC concentrations tested), employing MALDI-TOF MS we were able to detect proteomic changes in the presence of the antifungal.

Corroborating with previous reports, this work demonstrates the reduction of diagnosis time of AFST-MS (24-48 h vs. 15 h in this study). This is the main advantage of AFST-MS, considering that in cases of sepsis each hour delay in the appropriate antimicrobial administration is associated with an additional 7.6% mortality risk.^42^ A major advantage relates to the subjectivity reduction in result interpretation, which especially occurs when filamentous fungi are evaluated via BMD.^4^ ^,^ ^12^ ^,^ ^15^ Thus, our results indicate that AFST-MS is an alternative method that provides faster diagnosis when compared to conventional methods, which can potentially be implemented in routine laboratories in the future.

In conclusion, MALDI-TOF MS technology was demonstrated to be effective for AFST application in *Candida* and *Aspergillus* species and was shown to be a faster alternative when compared to traditional methods. Even though, more studies are necessary for optimisation and method standardisation for clinical routine application.

## References

[1] Vallabhaneni S, Mody RK, Walker T, Chiller T (2016). The global burden of fungal diseases. Infect Dis Clin North Am.

[2] Kim JY (2016). Human fungal pathogens Why should we learn?. J Microbiol.

[3] Perlin DS, Rautemaa-Richardson R, Alastruey-Izquierdo A (2017). The global problem of antifungal resistance prevalence, mechanisms, and management. Lancet Infect Dis.

[4] Roberto AEM, Xavier DE, Vidal EE, Vidal CFL, Neves RP, Lima-Neto RG (2020). Rapid detection of echinocandins resistance by MALDI-TOF MS in Candida parapsilosis Complex. Microorganisms.

[5] Brown GD, Denning DW, Gow NA, Levitz SM, Netea MG, White TC (2012). Hidden killers human fungal infections. Sci Transl Med.

[6] Fisher MC, Hawkins NJ, Sanglard D, Gurr SJ (2018). Worldwide emergence of resistance to antifungal drugs challenges human health and food security. Science.

[7] Sanglard D (2016). Emerging threats in antifungal-resistant fungal pathogens. Front Med.

[8] Alexander BD, Procop GW, Dufresne P, Fulller J, Ghannoum MA, Hanson KE (2012). Reference method for broth dilution antifungal susceptibility testing of yeasts.

[9] Knoll MA, Ulmer H, Lass-Flörl C (2021). Rapid antifungal susceptibility testing of yeasts and molds by MALDI-TOF MS a systematic review and meta-analysis. J Fungi (Basel).

[10] Saracli MA, Fothergill AW, Sutton DA, Wiederhold NP (2015). Detection of triazole resistance among Candida species by matrix-assisted laser desorption/ionization-time of flight mass spectrometry (MALDI-TOF MS). Med Mycol.

[11] Pfaller MA, Diekema DJ (2012). Progress in antifungal susceptibility testing of Candida spp by use of Clinical and Laboratory Standards Institute broth microdilution methods, 2010 to 2012. J Clin Microbiol.

[12] Posteraro B, Sanguinetti M (2014). The future of fungal susceptibility testing. Future Microbiol.

[13] Berkow EL, Lockhart SR, Ostrosky-Zeichner L (2020). Antifungal susceptibility testing current approaches. Clin Microbiol Rev.

[14] Oviaño M, Bou G (2018). Matrix-Assisted Laser Desorption Ionization-Time of Flight Mass Spectrometry for the rapid detection of antimicrobial resistance mechanisms and beyond. Clin Microbiol Rev.

[15] Delavy M, Dos Santos AR, Heiman CM, Coste AT (2019). Investigating antifungal susceptibility in Candida species With MALDI-TOF MS-based assays. Front Cell Infect Microbiol.

[16] Gitman MR, McTaggart L, Spinato J, Poopalarajah R, Lister E, Husain S (2017). Antifungal susceptibility testing of Aspergillus spp by using a Composite Correlation Index (CCI)-based Matrix-Assisted Laser Desorption Ionization-Time of Flight Mass Spectrometry method appears to not offer benefit over traditional broth microdilution testing. J Clin Microbiol.

[17] De Carolis E, Vella A, Florio AR, Posteraro P, Perlin DS, Sanguinetti M (2012). Use of matrix-assisted laser desorption ionization-time of flight mass spectrometry for caspofungin susceptibility testing of Candida and Aspergillus species. J Clin Microbiol.

[18] Vella A, De Carolis E, Vaccaro L, Posteraro P, Perlin DS, Kostrzewa M (2013). Rapid antifungal susceptibility testing by Matrix-Assisted Laser Desorption Ionization-Time of Flight Mass Spectrometry analysis. J Clin Microbiol.

[19] Balajee SA, Houbraken J, Verweij PE, Hong SB, Yaghuchi T, Varga J (2007). Aspergillus species identification in the clinical setting. Stud Mycol.

[20] Varga J, Frisvad JC, Samson RA (2011). Two new aflatoxin producing species, and an overview of Aspergillus section Flavi. Stud Mycol.

[21] Rex JH, Alexander BD, Andes D, Arthington-Skaggs B, Brown SD, Chaturveli V (2008). Reference method for broth dilution antifungal susceptibility testing of filamentous fungi; Approved Standard. CLSI.

[22] Rex JH, Alexander BD, Andes D, Arthington-Skaggs B, Brown SD, Chaturveli V (2008). Reference method for broth dilution antifungal susceptibility testing of yeasts; Approved Standard. CLSI.

[23] Arendrup MC, Friberg N, Mares M, Kahlmeter G, Meletiadis J, Guinea J, Subcommittee on Antifungal Susceptibility Testing of the ESCMID European Committee for Antimicrobial Susceptibility Testing (2020). How to interpret MICs of antifungal compounds according to the revised clinical breakpoints v 10.0 European committee on antimicrobial susceptibility testing (EUCAST). Clin Microbiol Infect.

[24] Espinel-Ingroff A, Barchiesi F, Cuenca-Estrella M, Pfaller MA, Rinaldi M, Rodriguez-Tudela JL (2005). International and multicenter comparison of EUCAST and CLSI M27-A2 broth microdilution methods for testing susceptibilities of Candida spp to fluconazole, itraconazole, posaconazole, and voriconazole. J Clin Microbiol.

[25] Pontes L, Beraquet CAG, Arai T, Pigolli GL, Lyra L, Watanabe A (2020). Aspergillus fumigatus clinical isolates carrying CYP51A with TR34/L98H/S297T/F495I substitutions detected after four-year retrospective azole resistance screening in Brazil. Antimicrob Agents Chemother.

[26] Prigitano A, Esposto MC, Grancini A, Passera M, Paolucci M, Stanzani M (2020). Prospective multicentre study on azole resistance in Aspergillus isolates from surveillance cultures in haematological patients in Italy. J Glob Antimicrob Resist.

[27] Peron IH, Reichert-Lima F, Busso-Lopes AF, Nagasako CK, Lyra L, Moretti ML (2016). Resistance surveillance in Candida albicans a five-year antifungal susceptibility evaluation in a Brazilian university hospital. PLoS One.

[28] Zadka H, Raykhshtat E, Uralev B, Bishouty N, Weiss-Meilik A, Adler A (2019). The implementation of rapid microbial identification via MALDI-ToF reduces mortality in gram-negative but not gram-positive bacteremia. Eur J Clin Microbiol Infect Dis.

[29] Yo CH, Shen YH, Hsu WT, Mekary RA, Chen ZR, Lee WJ (2022). MALDI-TOF mass spectrometry rapid pathogen identification and outcomes of patients with bloodstream infection a systematic review and meta-analysis. Microb Biotechnol.

[30] French K, Evans J, Tanner H, Gossain S, Hussain A (2016). The clinical impact of rapid, direct MALDI-ToF identification of bacteria from positive blood cultures. PLoS One.

[31] Alanio A, Beretti JL, Dauphin B, Mellado E, Quesne G, Lacroix C (2011). Matrix-assisted laser desorption ionization time-of-flight mass spectrometry for fast and accurate identification of clinically relevant Aspergillus species. Clin Microbiol Infect.

[32] Patterson TF, Thompson 3rd GR, Denning DW, Fishman JA, Hadley S, Herbrecht R (2016). Practice guidelines for the diagnosis and management of aspergillosis 2016 update by the Infectious Diseases Society of America. Clin Infect Dis.

[33] Berkow EL, Lockhart SR, Ostrosky-Zeichner L (2020). Antifungal susceptibility testing current approaches. Clin Microbiol Rev.

[34] Vandeputte P, Ferrari S, Coste AT (2012). Antifungal resistance and new strategies to control fungal infections. Int J Microbiol.

[35] Paul S, Singh P, Shamanth AS, Rudramurthy SM, Chakrabarti A, Ghosh AK (2018). Rapid detection of fluconazole resistance in Candida tropicalis by MALDI-TOF MS. Med Mycol.

[36] Marinach C, Alanio A, Palous M, Kwasek S, Fekkar A, Brossas JY (2009). MALDI-TOF MS-based drug susceptibility testing of pathogens the example of Candida albicans and fluconazole. Proteomics.

[37] Paul S, Singh P, Rudramurthy SM, Chakrabarti A, Ghosh AK (2017). Matrix-assisted laser desorption/ionization-time of flight mass spectrometry protocol standardization and database expansion for rapid identification of clinically important molds. Future Microbiol.

[38] Vella A, De Carolis E, Mello E, Perlin DS, Sanglard D, Sanguinetti M (2017). Potential use of MALDI-ToF Mass Spectrometry for rapid detection of antifungal resistance in the human pathogen Candida glabrata. Sci Rep.

[39] Delavy M, Cerutti L, Croxatto A, Prod'hom G.Sanglard D.Greub G (2020). Machine learning approach for Candida albicans fluconazole resistance detection using Matrix-Assisted Laser Desorption/Ionization Time-of-Flight Mass Spectrometry. Front Microbiol.

[40] De Carolis E, Marchionni F, La Rosa M, Meis JF, Chowdhary A, Posteraro B (2021). Are we ready for nosocomial Candida auris infections Rapid identification and antifungal resistance detection using MALDI-TOF mass spectrometry may be the answer. Front Cell Infect Microbiol.

[41] Yu SY, Guo LN, Xiao M, Zhou ML, Yuan Y, Wang Y (2020). Clinical and microbiological characterization of invasive pulmonary aspergillosis caused by Aspergillus lentulus in China. Front Microbiol.

[42] Kumar A, Roberts D, Wood KE, Light B, Parrillo JE, Sharma S (2006). Duration of hypotension before initiation of effective antimicrobial therapy is the critical determinant of survival in human septic shock. Crit Care Med.

